# Heroic viticulture: Environmental and socioeconomic challenges of unique heritage landscapes

**DOI:** 10.1016/j.isci.2023.107125

**Published:** 2023-07-14

**Authors:** Paolo Tarolli, Wendi Wang, Anton Pijl, Sara Cucchiaro, Eugenio Straffelini

**Affiliations:** 1 Department of Land, Environment, Agriculture and Forestry, University of Padova, Agripolis, Viale dell'Università 16, 35020 Legnaro, PD, Italy; 2 Cambisol consultancy, Rozenstraat 60, Veenendaal, the Netherlands; 3 Department of Agricultural, Food, Environmental and Animal Sciences, University of Udine, Italy

## Abstract

Steep-slope agricultural landscapes cover a small fraction of global agricultural areas.^1^ Despite the limited coverage, they are relevant for high-quality food and wine production, history, and landscape value. On steep slopes, centuries of effort and tradition have created a unique cultural heritage to be preserved. Here, peculiar traditional local knowledge of soil and water conservation combined with agronomic practices (e.g., dry-stone wall terracing) has been handed down for generations. However, such landscapes are fragile and under threat.

**Figure:**
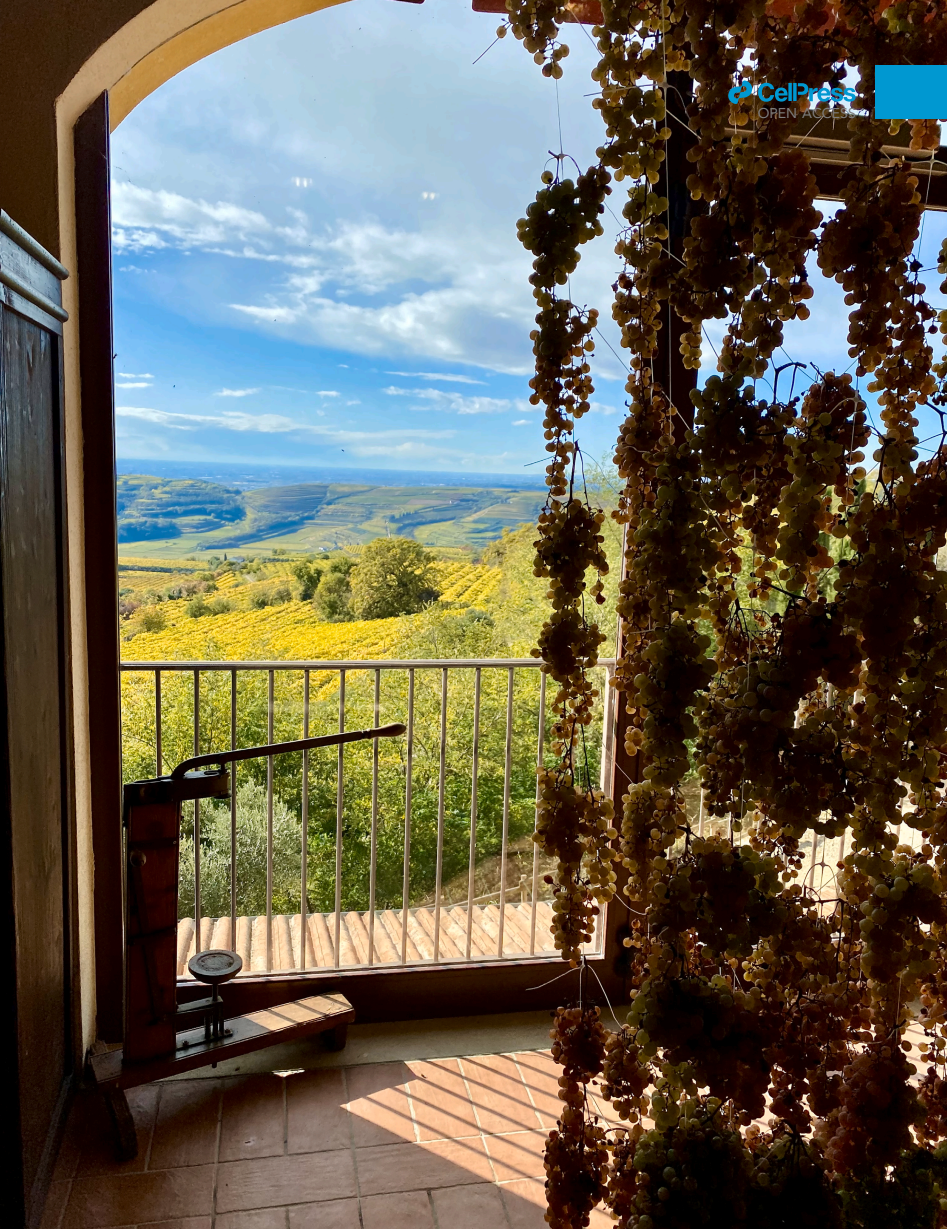
"Window view on Soave’s traditional vineyards cultivated on steep-slope landscapes and drying grapes using the *appassimento* process (Coffele farm, FAO-GIAHS site, North of Italy) (photo by P. Tarolli)"

“On steep slopes, centuries of effort and tradition have created a cultural heritage to be preserved.”

### Introduction

Viticulture is one of the most relevant agricultural systems of steep-slope landscapes. In Europe, we can find some of the most famous sites and popular wines (e.g., Port wine, Prosecco, Passito). Viticulture becomes “heroic” when practiced under extreme climatic, geomorphologic, and geographical conditions. Farmers are considered heroes because they deal with this “adverse” environment every day, typically by purely manual operations without the use of mechanized tools. Landscapes characterized by heroic viticulture have been recognized and protected by the United Nations, being inserted in the United Nations Educational, Scientific and Cultural Organization (UNESCO) World Heritage Sites and the Food and Agriculture Organization (FAO) Globally Important Agricultural Heritage Systems (GIAHS). Here, a virtuous combination of agronomic practice, famous wine, networks of restaurants, green tourism, and even religion and art (if churches are on the site) created an optimal condition for a circular economy and socioeconomic sustainability. Nevertheless, these landscapes are under threat by changing climate. The increased frequency of weather extremes driven by climate change accelerates soil degradation. Intense and localized rainfall events, if soil and water conservation solutions are not optimally adopted, can quickly trigger slope failures and widespread soil erosion processes on cultivated hillslopes. In addition, prolonged droughts, as we observed in Europe in 2022,^2^ could pose another criticality: sustainable water resources management on steep slopes. Managing water for irrigation on agricultural slopes greater than 50% (in extreme situations) is difficult; it costs and requires a very efficient and sustainable strategy. There is, however, a second criticality, which is the socioeconomic condition of our society. The last half past century has been characterized by rural exodus and a gradual abandonment of mountain landscapes. The new generation is not attracted to continue working under extreme conditions if economic benefits are insignificant. In addition, the technological modernization of society is “degrading” the rural cultural background of the previous generation. Because of the above critical threats, there is an urgent need to establish sustainable actions to keep people there and tradition alive. The risk is not only losing an agricultural product or seeing a landscape change, negatively impacting the local economy. The risk is losing entire communities' history and their cultural roots.

### What is steep slope or “heroic” viticulture, and why is it important?

Viticulture often finds ideal conditions on hillsides and mountainsides. There are many reasons, for example, optimal solar radiation, the correct day-night temperature fluctuation (essential for grape ripening), ventilation, and proper soil drainage. All these factors contribute to producing excellent and internationally recognized wines. Within the family of steep-slope viticulture, there is a narrow niche called "heroic viticulture". As already suggested by the nomenclature, this category is characterized by particularly complex environmental conditions, mainly due to high slope gradients or specific geomorphic features. CERVIM (Center for Research, Studies, and Enhancement for Mountain Viticulture) speaks of heroic viticulture when at least one of the following conditions can be observed: 1) slope greater than 30 percent; 2) altitude greater than 500 m above sea level; 3) vines are grown on agricultural terraces; and 4) vineyards are located on small islands.^3^ Europe is home to numerous heroic and, more in general, steep-slope vineyards. In particular, Italy has a unique heritage of such lands from north to south. Among the most famous vineyards are those of the Prosecco Hills of Conegliano and Valdobbiadene (“Colline del Prosecco di Conegliano e Valdobbiadene” UNESCO World Heritage Site), the "Soave Traditional Vineyards" (a FAO Globally Important Agricultural Heritage Systems site), or the traditional vineyards of Pantelleria Island (the agricultural practice of “vite ad alberello” - head-trained bush vines - is inscribed as UNESCO Intangible Cultural Heritage of Humanity). Other notable examples are the Portuguese Alto Douro region (UNESCO cultural landscape; Port wine production area) and the Spanish Canary Islands. Heroic vineyards are often the result of centuries of experience and tradition in grape growing and wine production. The survival of these crops depends on successfully implementing targeted interventions to manage water and soil, which are providers of vital ecosystem services. Agricultural terraces are the most widespread system in such landscapes, primarily needed to make extreme slope areas cultivable. They are also crucial for regulating water flow following intense rainfall, increasing plant water availability, limiting soil and (valuable) organic matter leaching, and mitigating hydrogeological risk. The great effort required to manage and survive these areas reinforces the specific human-environment connection. This is why they are recognized as cultural uniquenesses of primary historical and social importance, where traditional knowledge is the still determining element. Recognizing the value of these areas is the first step in designing protection and maintenance strategies. This is crucial from an economic and social point of view. In mountain areas, the market generated by heroic viticulture could significantly drive the local economy, discouraging the land abandonment of mountain communities.

### What is the relationship between local geology, the landscape, and viticulture?

Farming in extreme areas requires strong determination and equilibrium with the natural environment, the soil, and all the processes on its surface. Working on the agricultural terraces often means practicing traditional, manual viticulture with difficult support from mechanization due to limited accessibility to the fields. Achieving a balance with environmental conditions costs effort not only in preparing slopes for implementing soil and water conservation practices. It also requires constant care, in the form of continuous land maintenance, to prevent the consequences of extreme weather phenomena from causing land degradation. Slope steepness is the morphological parameter that determines the formation of surface runoff and its development on agricultural hillsides. It is capable of accumulating surface runoff water along preferential lines. If this occurs uncontrolled (e.g., without an appropriately sized drainage network), overland flow can cause soil erosion, terrace wall collapse, and landslide formation.^4^ This is a primary risk for viticulture as it can severely damage vineyard functionality and production, consequently requiring economical and human efforts in slope restoration. On the other hand, geology represents an addressed value to wine quality and the landscape’s uniqueness. The concept of “terroir”, widely used in viticulture, is highly influenced by this. Indeed, there are wine-growing areas where physical features (geology and soil properties, but also other aspects, such as climate) give wine characteristics that make it unique and recognizable to the consumer. Wine becomes a marketable product and an immersive experience in the land where it is produced. Calcareous soils are optimal for producing white wines that are elegant and low in tannins. In contrast, clay soils retain more organic matter, making the wine more structured, thus excellent for red wines. An interesting further example is volcanic soils. They are characterized by lava, ash, and basaltic deposits. According to specialists, volcanic soils help to characterize a wine and its intensity and structure, a condition that is impossible to find in other soils. Indeed,^5^ and are well-drained since the water tends to infiltrate quickly. Remarkable examples are volcanic islands (e.g., Canary, Madeira, Pantelleria), volcanoes (e.g., Etna), and specific sites where ancient volcanic activities occurred (e.g., Soave). There is a virtuous connection between landscape value, heritage, terroir, and wine quality in such landscapes.

## Viticulture and environmental change

### What are the main challenges heroic viticulture is facing?

At present, there are two major challenges that heroic viticulture is facing: intensification of weather extremes and socioeconomic sustainability. Climate change seriously threatens steep-slope agriculture.^1^ On the one hand, intense and localized rainfall events occurring on steep slopes (especially those affected by anthropogenic features such as roads or terraces) could trigger severe soil degradation and even mass movements. In such conditions, drainage systems should be adequately designed and maintained to manage excessive runoff, avoiding concentration on flow along preferential flow paths on rural roads. On the other hand, prolonged extreme drought conditions (e.g., the catastrophic drought in Europe in 2022^2^) create unprecedented criticalities for suitable water management. Irrigating a terrace system on slopes greater than 50% is difficult. Pumping the water from the plain or delivering water in tanks through trucks is economically and environmentally unsustainable. Excessive use of groundwater through the displacement of wells can be similarly unsustainable. The decrease in the groundwater table is a risk that could negatively impact the entire freshwater storage system in such complex landscapes. Here, and under such water scarcity conditions, traditional knowledge of rainwater harvesting could help. Microwater storage can offer sustainable and effective solutions. They have a low impact, and they are easy to design. If located adequately along the preferential flow path, they can collect rain from runoff events; that water then can be reused for emergency irrigation contributing to a more climate change-resilient agricultural system.^6^ In addition, they can create wetlands and refugees for birds and amphibians, contributing to biodiversity enrichment. Concerning socioeconomic conditions, the discussion is more complex, and adaptation strategies should involve policymakers and governments. If the economic benefits of having a farm in steep-slope areas are not significant, the risk of land abandonment is high. This condition will be worsened if extreme weather continues to increase. First, from the normative point of view, a system should be able to help those farms. Ad-hoc subsidies are welcomed, but there are other solutions. It is necessary to adopt clear non-structural measures and guidelines to protect such realities by offering competitive marketing networking (for some high-quality wines, this is already happening). Second, there is a need to pay more attention to education. Educating the new generation about the benefits of rural reality and the need to preserve cultural heritage, live in equilibrium with the environment, and have a sustainable approach to agriculture is not a negative fact. The value of viticulture can be far more than a mere bottle of wine and a form of monoculture. There are realities where the displacement of hedges and tree buffer zones and social farm activities (inside a wine farm) are virtuous examples of sustainable agriculture.

## Challenges and opportunities

### How can scientists and winemakers work together to address these challenges?

When facing complex and multifaceted issues like climate extremes or unsustainable agricultural practices, it is essential to take an integrated and multidisciplinary approach. The key to success lies in combining the traditional knowledge of winemakers with innovation and scientific rigor, along with targeted communication strategies to bring the scientific world closer to farmers and consumers. A first step could be defining scientific projects with high stakeholder involvement at all levels: from those involved in fieldwork to the wine technicians who care for the vines and vineyards, to those involved in wine production, to the marketing of the finished product. The latter can be intended as wine but also as an experience. Sustainable vineyard practices can also lead to green tourism, where the landscape becomes a vital component of the product. This approach can make the product unique and more attractive to consumers. Moreover, consumers are becoming increasingly aware of the different environmental challenges and investment in a more sustainable and safe territory. Technically, solutions for mitigating problems in the vineyard are diverse and can be grounded in three pillars: identifying critical areas, improving the sustainability of cultivation, and constant monitoring. Each of the activities can benefit from the combined work of farmers and scientific research in a mutualistic relationship where each actor compensates for possible shortcomings of the other. The technology can help identify critical areas, such as those most at risk of soil erosion, terrace wall collapse, surface landslides, and land degradation problems in general. The use of remote platforms as drones equipped with different types of payloads (such as laser scanners or cameras for photogrammetry) allows for very high-resolution 3D reconstruction of agricultural surfaces. If we combine these input data with geomorphological and hydrological analyses, detailed maps can be created to show farmers the areas of hydrogeologic risk and where to implement mitigation strategies. Nature-based solutions (NBSs) are strongly recommended as they can promote sustainably with green facilities, providing benefits and ecosystem services to human communities. With remote sensing costs becoming more affordable, these methods can find broad development within farms and help increase farming sustainability. For example, they can guide the choice of soil management to make vineyards more resilient to intense rainfall (such as using a permanent grass cover that prevents the detachment of soil particles) or drought (maintaining soil organic carbon content to improve water retention). They can even guide the implementation of soil and water conservation practices. An example is the case of rainwater storage facilities (that on steep slope they should be small in size), which can be optimally designed using high-resolution digital terrain models (DTMs) to mitigate surface runoff and collect water to be reused for irrigation in case of drought. Finally, monitoring is crucial for inspecting critical areas (such as observing alarming landslide movements) and for planning maintenance interventions to reduce hydrogeological risk. For instance, high-resolution digital models can be used to estimate the functionality of proposed interventions *a priori*, by assessing the effects of different types of soil management practices or drainage network designs. In this way, farms can work closely with scientists to optimize investments for a more functional, sustainable, and safe agricultural landscape—a winning alliance to face these diverse natural and anthropogenic challenges.

**Figure:**
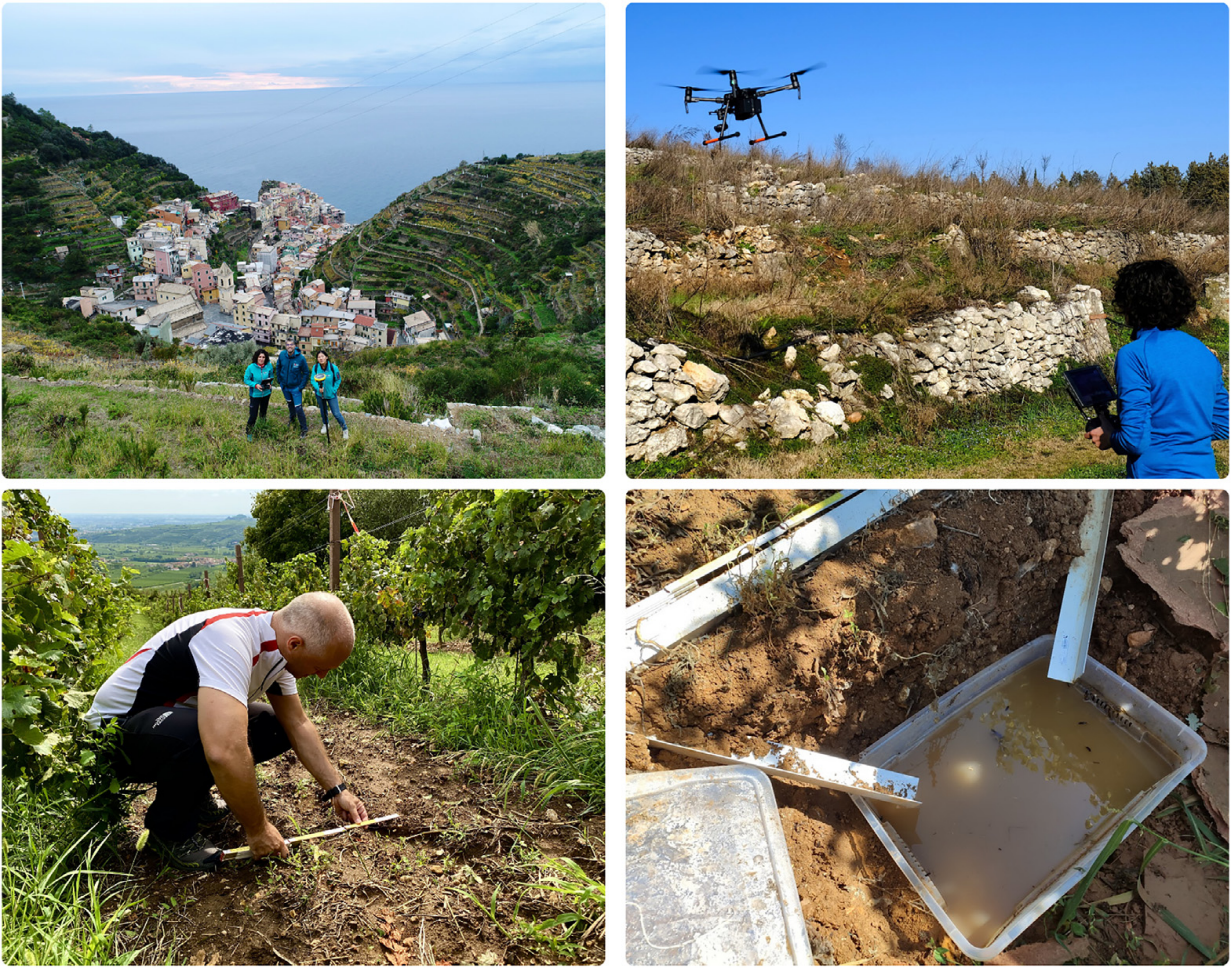
Drone view on Manarola (Cinque Terre) dry-stone wall terraced landscape (UNESCO site and National Park); drone survey of an abandoned terrace system and soil erosion monitoring in Soave vineyard landscape (GIAHS site). (Photographs by S. Cucchiaro, E. Straffelini, P. Tarolli)
